# Electronic Health Record-Based Surveillance for Community Transmitted COVID-19 in the Emergency Department

**DOI:** 10.5811/westjem.2020.5.47606

**Published:** 2020-05-22

**Authors:** Michael S. Pulia, Daniel J. Hekman, Joshua M. Glazer, Ciara Barclay-Buchanan, Nicholas Kuehnel, Joshua Ross, Brian Sharp, Robert Batt, Brian W. Patterson

**Affiliations:** * University of Wisconsin – Madison, BerbeeWalsh Department of Emergency Medicine, Madison, Wisconsin; † University of Wisconsin – Madison, Department of Operations and Information Management, Madison, Wisconsin

## Abstract

**Introduction:**

SARS-CoV-2, a novel coronavirus, manifests as a respiratory syndrome (COVID-19) and is the cause of an ongoing pandemic. The response to COVID-19 in the United States has been hampered by an overall lack of diagnostic testing capacity. To address uncertainty about ongoing levels of SARS-CoV-2 community transmission early in the pandemic, we aimed to develop a surveillance tool using readily available emergency department (ED) operations data extracted from the electronic health record (EHR). This involved optimizing the identification of acute respiratory infection (ARI)-related encounters and then comparing metrics for these encounters before and after the confirmation of SARS-CoV-2 community transmission.

**Methods:**

We performed an observational study using operational EHR data from two Midwest EDs with a combined annual census of over 80,000. Data were collected three weeks before and after the first confirmed case of local SARS-CoV-2 community transmission. To optimize capture of ARI cases, we compared various metrics including chief complaint, discharge diagnoses, and ARI-related orders. Operational metrics for ARI cases, including volume, pathogen identification, and illness severity, were compared between the preand post-community transmission timeframes using chi-square tests of independence.

**Results:**

Compared to our combined definition of ARI, chief complaint, discharge diagnoses, and isolation orders individually identified less than half of the cases. Respiratory pathogen testing was the top performing individual ARI definition but still only identified 72.2% of cases. From the pre to post periods, we observed significant increases in ED volumes due to ARI and ARI cases without identified pathogen.

**Conclusion:**

Certain methods for identifying ARI cases in the ED may be inadequate and multiple criteria should be used to optimize capture. In the absence of widely available SARS-CoV-2 testing, operational metrics for ARI-related encounters, especially the proportion of cases involving negative pathogen testing, are useful indicators for active surveillance of potential COVID-19 related ED visits.

## INTRODUCTION

SARS-CoV-2, a novel coronavirus, is the cause of an ongoing global pandemic. It can cause a serious respiratory illness, termed COVID-19, with comorbid and older adults at increased risk of death.^1^,^2^ While other affected countries instituted widespread testing for SARS-CoV-2 as part of early and successful mitigation efforts, due to a variety of factors, diagnostic testing efforts in the United States (US) during early phases of community spread continue to be significantly hampered.^3^,^4^ The lack of testing capacity resulted in stringent testing recommendations from the Centers for Diseases Control and Prevention, which specifically excluded asymptomatic or mildly symptomatic individuals. The delay in community-based surveillance has generated substantial uncertainty among health systems attempting to prepare for a surge in cases and severely limited a primary tool of pandemic mitigation: source identification and contract tracing. Early detection of a surge in emergency department (ED) COVID-19 cases is essential to guide response plans if hospitals hope to avoid overwhelmed systems.

Therefore, faced with the absence of a readily available rapid diagnostic assay, we developed a simple, electronic health record (EHR)-based tracking tool to detect variations in encounters due to acute respiratory infections (ARI), organism identification for ARIs, or ARI acuity related to potential unrecognized COVID-19 community transmission. The first step was to develop a process for identifying ARI using available EHR data. The second step was to determine whether we could detect a significant change in ARI case without identified pathogen as a metric of potential COVID-19 community transmission.

## METHODS

We conducted this project using combined EHR data from an academic medical center ED with over 60,000 patient visits per year and an affiliated community ED with over 20,000 annual visits. Data were collected for ED arrivals from February 17-March 30, 2020, which included three weeks before and after confirmed local SARS-CoV-2 transmission. All ED visits during this time period were included in our dataset and examined for potential ARI. SARS-CoV-2 testing was available via the state department of health and later in-house during the post three-week time period but for select patient groups only.^5^ All data were electronically extracted from the EHR by an experienced data analyst. This project was considered quality improvement and did not meet the federal definition of human subject research pursuant to 45 CFR 46 as assessed using a self-certification tool provided by our institutional health sciences institutional review board.

Using the consensus of our departmental COVID-19 response team, including ED operations, informatics, and infectious diseases experts, we identified four potential EHR-based criteria to identify ARI encounters and a series of operational metrics for inclusion in an ARI outbreak tracking tool. Each selected metric needed to satisfy two basic criteria: 1) readily extractable electronically from existing EHR data; 2) involve only simple calculations for ease of interpretation and translation to other EHR platforms. For the first metric, overall ARI volume, we applied four criteria to all ED encounters to determine what would provide the most comprehensive capture of potential cases: 1) chief complaints specific to ARI (cough, flu-like symptoms, sore throat, upper respiratory symptoms, sinus symptoms); 2) discharge diagnoses specific to ARI (ICD-10 codes J00–J06, J09–J18, J20–J22, J40); 3) respiratory pathogen isolation order; and 4) respiratory pathogen test order (influenza/respiratory syncytial virus assay, group A streptococcus swab, expanded viral polymerase chain reaction [PCR] panel).

Given the disproportionate rate of critical illness among patients with COVID-19 as compared to other ARI (e.g., seasonal influenza), we then included three metrics of severity: 1) percentage of patients with ARI requiring admission; 2) percentage of patients with ARI admitted to intermediate care or intensive care units (IMC/ICU); and 3) percentage of patients with ARI receiving antibacterial therapy. The antibiotic metric was included to capture any increase in ARI patients being treated with empiric antibiotics (e.g., met sepsis criteria). Finally, given the restricted testing criteria in place and ongoing uncertainty about the SARS-CoV-2 RNA PCR assay’s sensitivity and specificity^6^ to identify ARI encounters, potentially due to undiagnosed COVID-19, we selected a metric of percent ARI without an identified pathogen on any organism identification assay. This was selected due to the lack of discriminating clinical features between influenza and COVID-19 and the ongoing routine use of influenza/respiratory syncytial virus (RSV), expanded viral panel and group A strep assays for ARIs in our ED. Data were extracted from the EHR and analyzed using R 3.6.2 (The R Project for Statistical Computing, CRAN). We compared proportions of encounters before and after community transmission using a chi-square test.

## RESULTS

The combined overall ED census in our two departments decreased from 5213 to 3550 (−1663 encounters) from the preto post-time period, but the proportion of ED visits due to ARI increased significantly (6.6%, 95% confidence interval 4.6–8.5%, p<.001). When identifying ARI cases, we first created a combination definition using the union of all four individual criteria and applied it to all ED encounters. This identified 2540 total ARI encounters over the six-week period. When examined individually, each of the four criteria identified unique ARI cases (Figure). Specifically, when compared to the combined definition, chief complaint specific to ARI identified 32.7% of cases while discharge diagnosis related to ARI identified 42.4% of cases. Orders for respiratory pathogen isolation or respiratory pathogen testing identified 33.7% and 72.2% of cases, respectively.

The Table  compares our selected ED ARI metrics from three weeks before and three weeks after the local onset of community SARS-CoV-2 transmission. ARI encounters without an identified pathogen increased despite no change in the proportion of ARI encounters receiving pathogen testing. Of note, only 40% of ARI encounters received SARS-CoV-2 testing in the post period with a positivity rate of 6% (27/462 tested). In terms of acuity metrics, we did not detect a statistically significant change in overall ARI admissions or those requiring IMC/ICU care. There was a statistically significant decline in the proportion of ARI cases receiving antibiotics.

## DISCUSSION

In the early stages of the COVID-19 pandemic, we developed an ED surveillance tool for ARI encounters potentially related to undiagnosed community transmitted SARS-CoV-2 using readily available EHR data and simple calculations. As such, this tool could be easily implemented at other institutions. This approach to surveillance would especially benefit hospitals that do not currently have access to rapid SARS-CoV-2 identification assays or those adhering to restricted COVID-19 testing criteria as part of efforts to preserve personal protective equipment (PPE) or testing capacity. Despite rapid progress in available diagnostics, ongoing concerns over PPE and reagent shortages will continue to hamper widespread community surveillance testing in the US.^7^

Our approach expands upon more basic methods for ED-based seasonal influenza surveillance efforts by evaluating a multi-component definition of ARI that combines chief complaints and discharge diagnoses with actual orders for respiratory isolation and pathogen testing. Our results suggest the traditional approach of using chief complaint (e.g., influenza-like illness [ILI]) and/or discharge diagnoses alone may be inadequate for comprehensive identification of ARI encounters.^8^ Of note, we did exclude fever alone in our chief complaint definition as it is not specific to ARI and would result in capture of many infections unrelated to COVID-19 (e.g., urinary tract and skin infections).

In the case of a respiratory pandemic due to a novel pathogen, traditional, laboratory-based surveillance will also be ineffective. Based on ongoing influenza activity during the study time period and its similar clinical presentation to COVID-19, we selected percentage of ARI cases receiving pathogen testing with negative results as our metric for potential cases of undiagnosed COVID-19. The observed significant increase in overall ARI encounters and those without identified pathogen mirrors national observations of increased encounters for ILI without identified pathogen over the same time periods.^9^–^11^ This late-season spike in ILI cases, which did not occur in previous years, was confirmed in one report from Los Angeles to partially represent community transmission of COVID-19. Among patients with ILI who were tested for SARS-CoV-2, they observed a 5% positivity rate which is similar to our findings (6% positivity), suggesting a similar community transmission burden in disparate geographic locations around the same time period.^11^ Although we did not demonstrate a difference in our markers of ARI severity, we attribute this to continued low volumes of COVID-19 cases in our community. We anticipate these metrics will become increasingly valuable for early identification of a need for additional intensive care resources should ongoing mitigation efforts not succeed in flattening the outbreak curve locally.

## LIMITATIONS

For ARI case identification, it is possible that cases that would have been identified with manual chart review were excluded. Given the dynamic nature of the ongoing COVID-19 pandemic and limited post-community spread data available for analysis, we did not perform a formal, interrupted time series analysis to account for temporal effects. ARI volumes and percentage of ARI cases without identified pathogen must be interpreted based on local outbreak dynamics. As population-level surveillance metrics, these indicators should not be used to inform diagnosis or treatment decisions for individual patients.

## CONCLUSION

In this project, we evaluated a strategy for using EHR data to identify ARI-related ED encounters and demonstrated significant changes in metrics related to these encounters during the onset of local community SARS-CoV-2 transmission. ARI without identified pathogen encounters may serve as a lead population-level surveillance indicator for ARI outbreaks related to novel pathogens, such as the ongoing COVID-19 pandemic.

## Figures and Tables

**Figure:**
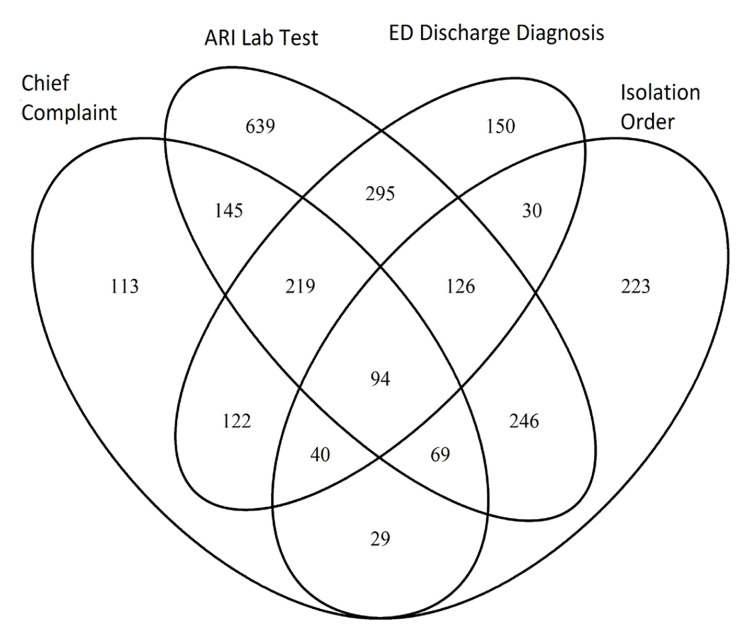
Number of acute respiratory infection encounters identified by different criteria. *ARI*, acute respiratory infections; *ED*, emergency department.

**Table:** Acute respiratory infection-related emergency department encounter metrics before and after community transmission of SARS-CoV-2.

	Pre-Community Transmission (n = 1372)	Post-Community Transmission (n = 1168)	Difference in Proportions (%, 95% CI)	P value
Pathogen Testing	998 (72.7% )	835 (71.5% )	−1.2% (−4.8%,2.2%)	0.512
Negative Pathogen Test	578 (42.1%)	642 (55.0%)	12.9% (9%,16.7%)	**<0.001**
Admitted	412 (30.0%)	374 (32.0%)	2% (−1.6%,5.6%)	0.299
Admitted to IMC/ICU	79 (5.8%)	83 (7.1%)	1.3% (−0.6%,3.3%)	0.192
Antibiotic Use	245 (17.9%)	165 (14.1%)	−3.8% (−6.6%,−0.9%)	**0.001**

*CI*, confidence interval; *IMC*, intermediate care unit; *ICU*, intensive care unit.
